# Spectrum of infection and outcomes in individuals with *Candida auris* infection in Qatar

**DOI:** 10.1371/journal.pone.0302629

**Published:** 2024-05-23

**Authors:** Jameela A. Al Ajmi, Aimon B. Malik, Hanaa Nafady-Hego, Fathima Hanana, Joji Abraham, Humberto G. Garcell, Ghada Hudaib, Walid Al-Wali, Faiha Eltayeb, Sherin Shams, Anil G. Thomas, Samah Saleem, Abdul-Badi Abou-Samra, Adeel A. Butt

**Affiliations:** 1 Corporate Quality and Patient Safety Department, Hamad Medical Corporation, Doha, Qatar; 2 Microbiology and Immunology, Faculty of Medicine, Assiut University, Assiut, Egypt; 3 Infection Prevention and Control Department, The Cuban Hospital, Dukhan, Qatar; 4 Department of Microbiology and Laboratory Medicine, Hamad Medical Corporation, Doha, Qatar; 5 Department of Medicine and Population Health Sciences, Weill Cornell Medicine, New York, NY, United States of America; 6 Department of Medicine and Population Health Sciences, Weill Cornell Medicine, Education City, Qatar; Shiraz University of Medical Sciences, ISLAMIC REPUBLIC OF IRAN

## Abstract

**Background:**

We investigated the spectrum of infection and risk factors for invasive fungal disease due to *Candida auris* (CA) in Qatar.

**Methods:**

We performed structured chart reviews on individuals with any positive CA culture between May 2019 and December 2022 at three tertiary care hospitals in Qatar. Invasive CA disease (ICAD) was defined as a positive sterile site culture, or any positive culture for CA with appropriate antifungal prescription. Main outcomes included proportion of individuals who developed ICAD among those with positive cultures, and 30-day/in-hospital mortality.

**Results:**

Among 331 eligible individuals, median age was 56 years, 83.1% were male, 70.7% were non-Qataris, and 37.5% had ≥ 3 comorbidities at baseline. Overall, 86.4% were deemed to have colonization and 13.6% developed ICAD. Those with ICAD were more likely to have invasive central venous or urinary catheterization and mechanical ventilation. Individuals with ICAD had longer prior ICU stay (16 vs 26 days, *P = 0*.*002*), and longer hospital length of stay (63 vs. 43 days; *P = 0*.*003*), and higher 30-day mortality (38% vs. 14%; *P<0*.*001*). In multivariable regression analysis, only mechanical ventilation was associated with a higher risk of ICAD (OR 3.33, 95% CI 1.09–10.17).

**Conclusion:**

Invasive *Candida auris* Disease is associated with longer hospital stay and higher mortality. Severely ill persons on mechanical ventilation should be especially monitored for development of ICAD.

## Introduction

*Candida auris* (CA) was first isolated from the ear canal of an inpatient in a hospital in Japan and has since become a global problem [1]. CA is often transmitted from person-to-person in the hospital setting and outbreaks have been reported across the globe [2]. Its association with healthcare outbreaks, difficulty in laboratory identification using traditional laboratory techniques, ability to form biofilms, ability to persist on inanimate surfaces and equipment for weeks, and multi-drug resistance, pose additional significant challenges in management of outbreaks and infection [3]. Invasive and bloodstream CA infections have been reported in nosocomial outbreaks, particularly in intensive care units (ICUs) [4]. Risk factors for invasive CA disease are generally the same as those for other *Candida* species and include multiple medical comorbidities, immunocompromised status, neutropenia, invasive devices, mechanical ventilation, ICU stay, and prolonged use of antibiotics [2].

Outbreaks of CA have been reported from the Middle East [5–10]. The genomic epidemiology of CA during an outbreak in Qatar was previously reported and revealed that all isolated belonged to the South Asian lineage and previous acquisition from foreign healthcare [11–13]. Repeated outbreaks in Qatar are an area of concern that need to be addressed. *Candida auris* is a highly resistant yeast and outbreaks have been associated with increased morbidity and mortality [4]. In a previous report, sequencing of simultaneous environmental isolates suggested a link to infected/colonized patients and the hospital environment. Incidence and risk factors for development of invasive *C*. *auris* disease among those with positive surveillance or routine cultures is not well defined. Here, we report the spectrum of infection and outcomes in individuals with CA infection in Qatar [11].

## Methods

### Study population and study setting

We conducted a retrospective study at three tertiary care hospitals in Qatar from May 2019 to December 2022. These hospitals are a part of Hamad Medical Corporation (HMC) network, Qatar, which is the public sector healthcare system providing >85% of the national inpatient bed capacity in Qatar. All HMC hospitals are accredited by the Joint Commission International (JCI, Chicago, IL, USA) and use the same electronic medical records platform (Cerner, Kansas City, MO, USA). All three hospitals are tertiary care facilities with a combined inpatient capacity of 945 beds (603, 267, and 75 beds respectively). All individuals with a positive CA culture within the study dates were identified from microbiology laboratory records. All analyzed clinical specimens were processed according to laboratory standard operative protocol at each microbiology laboratory, which are accredited by the College of American Pathologists. *C*. *Auris* from blood, urine, pleural fluid samples, were cultured on Sabouraud Dextrose Agar (Oxoid, UK) and Chromogenic Candida Agar (Oxoid, UK) and incubated at 35–37°C for 48 hours. Preliminary *C*. *auris* strain identification was based on colony morphology on Chromogenic Candida Agar (OXOID, UK), while the identification to species level was either confirmed by VITEK 2XL automated system (BioMerieux) or MALDI-TOF according to the manufacturer’s protocol of partial extraction. For screening patients for *C*. *Auris*, molecular detection by PCR was performed using AurisID (OLM Diagnostics, Canada).

A detailed chart review was performed using a structured data collection form using uniform definitions. Demographic, clinical, pharmacy and relevant microbiology/laboratory data were reviewed. Additional data reviewed included intensive care unit stay, use of central venous catheters, urinary catheters, mechanical ventilation, invasive surgical procedures, and vital status of the individuals. Based on current practice in our hospitals, a positive CA culture was categorized as colonization if the reason for testing was listed as surveillance, and the testing site was axilla, groin, or skin, and there were no clinical signs of infection. Invasive CA disease (ICAD) was defined as a positive sterile site culture (blood, catheter tip, joint fluid, cerebrospinal fluid), or a positive respiratory secretion, urine, or wound culture in individuals with a physician note documenting CA disease plus prescription of an appropriate antifungal agent for CA. This definition closely mirrors the definition of ICAD proposed by the European Organization for Research and Treatment of Cancer and the Mycoses Study Group Education and Research Consortium through a consensus conference [14]. Catheters, mechanical ventilation, intensive care unit stay, and antibiotic use were deemed to be a contributing risk factor if they were present within 7 days prior to the first positive culture. For those with multiple positive cultures, the date of the first positive culture was used as the index date.

### Outcome measures

The main outcomes include the proportion of individuals who developed ICAD, and 30-day or in-hospital mortality in individuals with colonization vs. those with invasive disease.

### Statistical analyses

Baseline characteristics were compared using the chi-squared or the t-test as appropriate. Mean (for normally distributed data) or median (for the non-normally distributed data) were used to summarize the data, along with the standard deviation or inter-quartile range to demonstrate the spread. Kaplan-Meier curves were used to demonstrate the difference in survival among those with and without invasive disease. Time at risk started from the date of index positive culture date for CA.

### Ethics approval and consent to participate

The study was approved by the Institutional Review Board (IRB) at Hamad Medical Corporation (Study ID MRC-01-23-202). A waiver of informed consent was granted by the IRB due to the retrospective nature of data collection. The period of data collection spanned from January 1, 2020, to December 31, 2022. The data were accessed for research purposes on June 01, 2023. Only approved study team members in this protocol had access to the data. After the creation of the analysis database, investigators did not have access to individual participants’ identifying information.

## Results

A total of 331 individuals had at least one positive CA culture during the study period. The median age was 56 years (IQR 44,70), 83.1% were male, and 70.7% were non-Qatari nationals **(Table 1).** Overall, 18.4% had no comorbidity, and 37.5% had ≥ 3 comorbidities at baseline. Other baseline characteristics are presented in **Table 1**. Forty-five individuals (13.6%) developed invasive *C*. *auris* disease (ICAD). While the burden of comorbidities was similar among those who developed ICAD compared to those who did not, those with ICAD were more likely to have a central venous catheter or a urinary catheter and were more likely to be on mechanical ventilation in the 7 days prior to the positive index culture. Antibiotic use in the previous 7 days was high and similar in both groups (84–87%). While a similar proportion of individuals were admitted to the ICU in the 7 days preceding the index positive culture, the median duration of such ICU stay was longer in those who developed ICAD 26 days (IQR 18.5, 42.5) compared to those without ICAD 16 days (IQR 9.0, 32.0; *P = 0*.*002*) **(Table 1).** The most common culture sites among 45 individuals with ICAD were blood (28.9%), urine (28.9%), and respiratory tract (13.3%). Eight individuals (27.8%) had >1 sterile site culture positive for CA.

**Table 1:** Baseline characteristics of individuals with *C*. *auris* colonization and invasive disease.

	Total (N = 331)	Colonization (N = 286)	Invasive disease (N = 45)	P value
	N (%)	N (%)	N (%)	
Median age (IQR) years	56.0 (44.0–70.0)	56.0(43.0, 70.0)	43.0 (22.0, 92.5)	0.98
Sex				0.79
Female	56 (16.9)	49 (17.1)	7 (15.6)	
Male	275 (83.1)	237 (82.9)	38 (84.4)	
Nationality				0.68
Qatari	97 (29.4)	85 (29.7)	12 (26.7)	
Non-Qatari	234 (70.7)	201 (70.3)	33 (73.3)	
Comorbidities				0.76
None	61 (18.4)	54 (18.9)	7 (15.6)	
1–2	146 (44.1)	124 (43.4)	22 (48.9)	
≥3	124 (37.5)	108 (37.8)	16 (35.6)	
Hypertension	164 (49.5)	139 (48.6)	25 (55.6)	0.39
Diabetes mellitus	165 (49.8)	143 (50.0)	22 (48.9)	0.89
Chronic respiratory disease	47 (14.2)	42 (14.7)	10.6 (11.1)	0.52
Chronic kidney disease	64 (19.3)	53 (18.5)	11 (24.4)	0.35
Chronic liver disease	18 (5.4)	16 (5.6)	2 (4.4)	0.75
Cardiovascular disease	53 (16.0)	46 (16.1)	7 (15.6)	0.93
Cardiovascular disease	9 (2.7)	7 (2.4)	2 (4.4)	0.44
Solid organ Transplant	34 (10.3)	26 (9.1)	8 (17.8)	0.07
Others	132 (39.9)	116 (40.6)	16 (35.6)	0.52
Central Venous Catheter*	162 (48.9)	130 (45.5)	32 (71.1)	0.001
Urinary Catheter *	159 (48.0)	128 (44.8)	31 (68.9)	0.003
Mechanical ventilation*	208 (62.8)	169 (59.1)	39 (86.7)	<0.001
Anti-microbial therapy*	279 (84.3)	240 (83.9)	39 (86.7)	0.64
ICU stay*	283 (85.5)	242 (84.6)	41 (91.1)	0.25
Duration of ICU stay prior to index positive culture, Median (IQR) days	19 (10.0,33.0)	16.0 (9.0, 32.0)	26.0 (18.5, 42.5)	0.002
Total hospital stay, Median (IQR) days	43.0 (23.0, 93.0)	43.0 (22.0, 92.5)	63.0 (37.0, 192.0)	0.003
Deaths within 30 days of index positive culture	57 (17.2)	40 (14.0)	17 (37.8)	<0.001

* Within 7 days prior to the index positive culture.

ICU, intensive care unit; IQR, inter-quartile range.

The overall median hospital length of stay for the admission with the index positive culture was higher among those who developed ICAD (63 days, IQR 37, 192) compared with those with CA colonization only (43 days, IQR 22, 92; p-value 0.003) **(Table 1).** Proportion of deaths within 30 days of the index positive culture was similarly higher among those with ICAD compared with those with CA colonization only (37.8% vs. 14.0%, p<0.001) **(Table 1)**.

**Fig 1:**
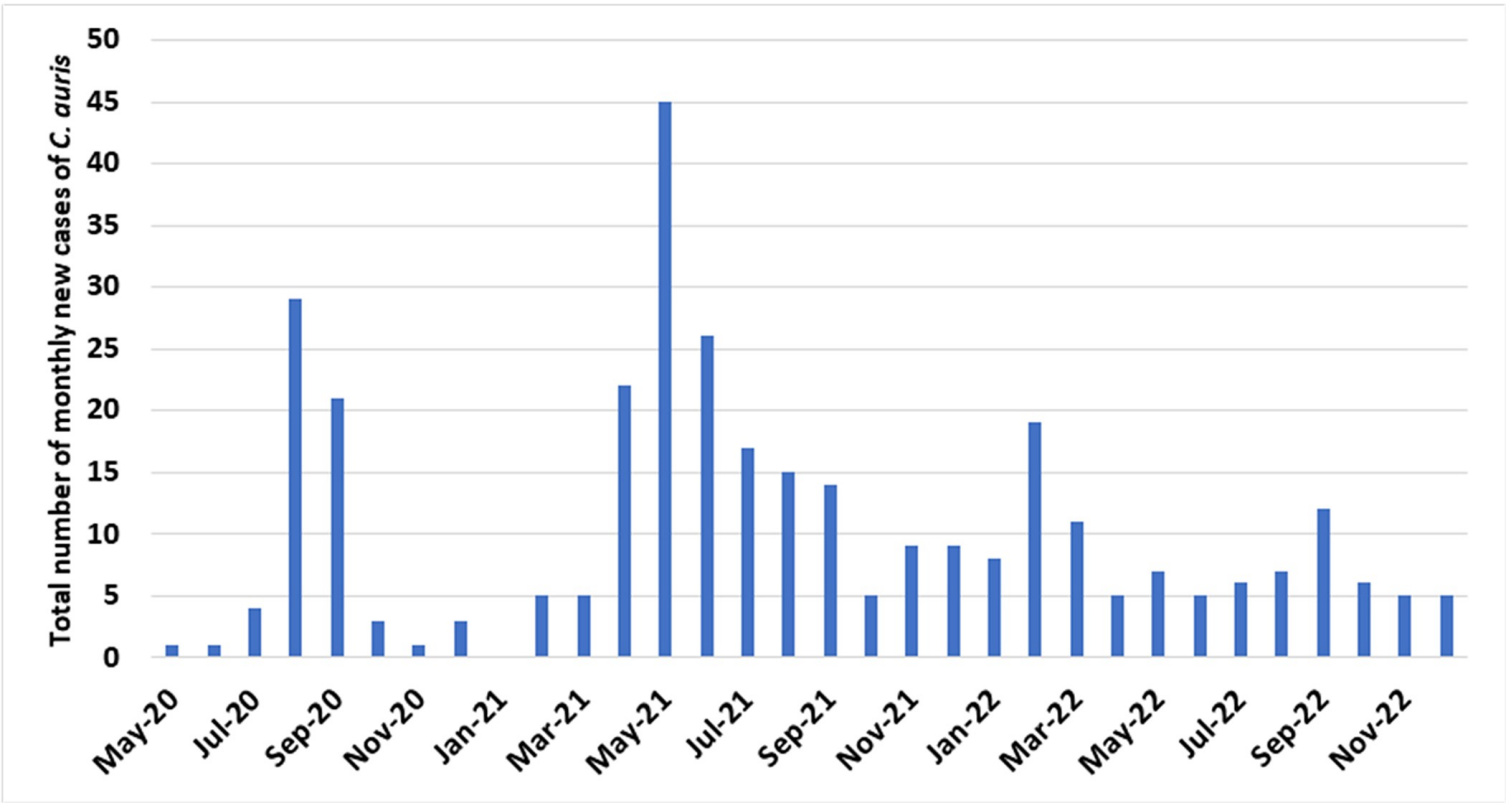
Total number of monthly new isolates of *C*. *auris* identified during the study period.

**Fig 2:**
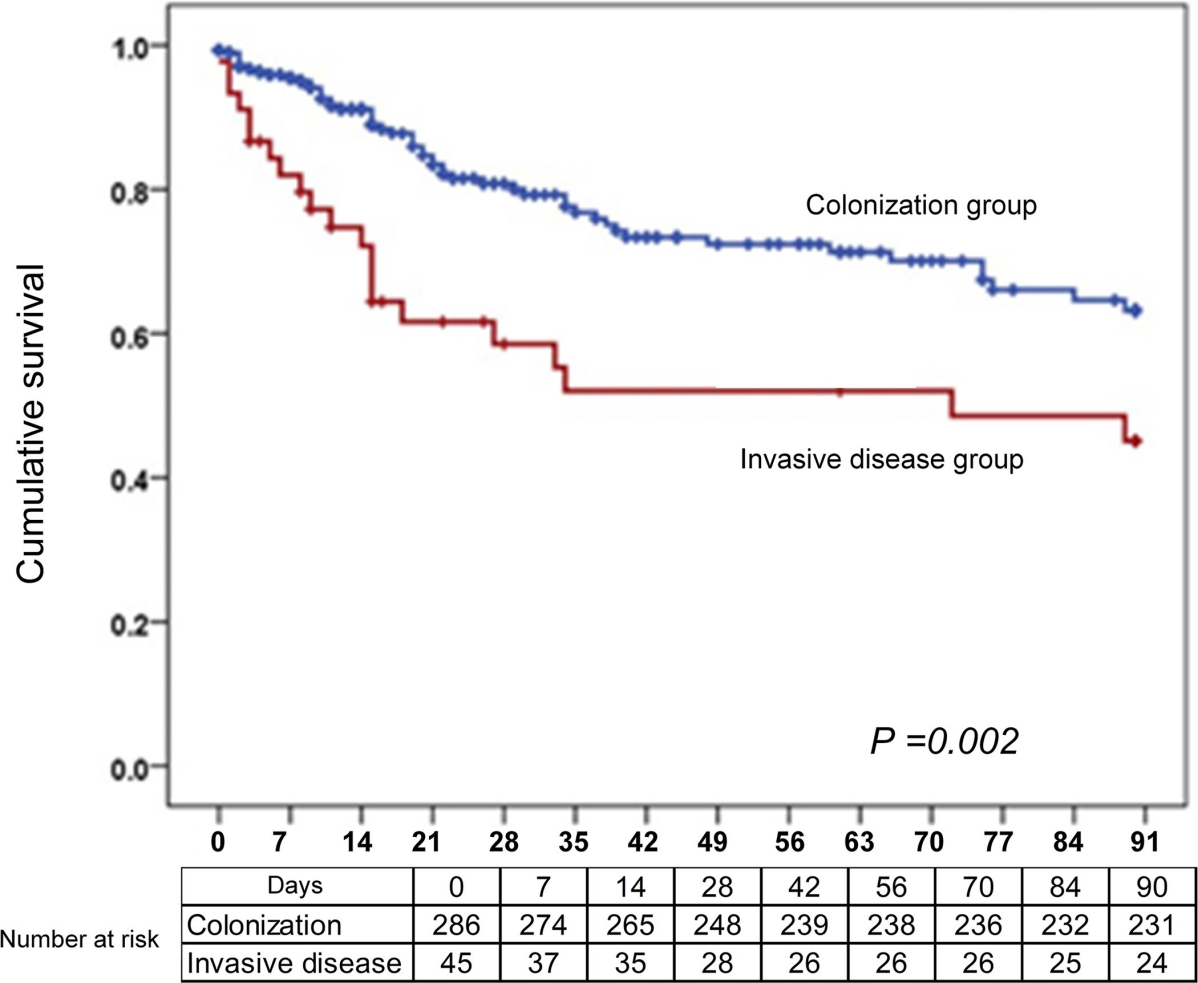
Kaplan–Meier curves demonstrating the survival of individuals with *C*. *auris* colonization and invasive disease. (Log-rank P-value = 0.002).

The temporal occurrence of cases is shown in **Fig 1**. There were two larger peaks in approximately mid-2020 and mid-2021, and two smaller peaks in early and late 2022. Kaplan-Meier curves demonstrated a significantly higher mortality among persons who had developed ICAD compared with those who did not **(Fig 2).** In multivariable regression analysis, mechanical ventilation in the preceding 7 days (adjusted OR 3.33, 95% CI 1.09–10.17) and cancer diagnosis (adjusted OR 4.02, 95% CI 1.41–11.45) were associated with a higher risk of developing invasive disease **(Table 2).** Placement of a central venous catheter or a urinary catheter were associated with a higher risk in univariate analysis but were not significant in the multivariate analysis (P = 0.001 for both).

**Table 2:** Factors associated with development of *Candida auris* invasive fungal disease among those with positive cultures (Cox regression analysis).

	OR (95% CI)	p-value
Age per10 year increase	1.04 (0.80–1.34)	0.78
Male sex	1.33 (0.51–3.47)	0.56
Non-Qatari nationality	1.42 (0.59–3.43)	0.44
Comorbidities		
Hypertension	1.42 (0.66–3.06)	0.37
Diabetes mellitus	0.70 (0.33–1.48)	0.35
Chronic respiratory disease	0.45 (0.14–1.45)	0.18
Chronic kidney disease	1.73 (0.68–4.48)	0.26
Chronic liver disease	0.54 (0.10–2.82)	0.47
Cardiovascular disease	1.10 (0.36–3.04)	0.93
Solid organ Transplant	2.33(0.38–14.31)	0.36
Cancer diagnosis	4.02 (1.41–11.45)	0.01
Intensive care unit stay*	1.58 (0.47–5.35)	0.46
Central Venous Catheter*	0.78 (0.24–2.55)	0.68
Urinary Catheter*	2.07 (0.72–5.89)	0.18
Mechanical ventilation*	4.64 (1.43–15.05)	0.01
Antibiotic therapy*	1.18 (0.40–3.50)	0.77

* In the 7 days prior to index positive culture date.

## Discussion

Our study offers valuable insights into the prevalence and risk factors associated with Invasive *Candida auris* Disease (ICAD) in a large cohort of 331 individuals at multiple hospitals. The clinical course of these individuals was carefully reviewed to identify patterns and potential predictors of ICAD.

Several risk factors predispose hospitalized patients to invasive fungal disease, including invasive devices, length of stay in the intensive care unit, prescription of broad spectrum antibiotics, receipt of total parenteral nutrition and certain underlying comorbidities [15,16]. Previously reports suggest that up to 25% of critically ill patients colonized with CA develop candidemia [4]. In our study, only a small proportion (13.6%) of individuals with a positive CA culture developed ICAD during the study period. Cancer diagnosis and mechanical ventilation were the only independent risk factors identified for development of ICAD. While these are widely accepted risk factors for invasive fungal disease, other traditional factors were not associated with a higher risk. There are at least two possible reasons for this observation. Firstly, some traditional risk factors, e.g. antibiotic use was nearly universally observed in both groups precluding a robust analysis of this factor as a contributor towards ICAD. Secondly, some other traditional risk factors, e.g. invasive catheters, may just be a marker of severe disease which is also reflected by the use of mechanical ventilation. Regardless, it is clear that among individuals with positive CA cultures, those with severe underlying disease are at a higher risk of developing ICAD.

The temporal trends of positive CA cultures exhibited two larger peaks in mid-2020 and mid-2021, along with two smaller peaks in early and late 2022. A previous report found low genetic variability during the first two peaks, indicating a clonal outbreak [12]. This suggests that the dynamics of CA outbreaks in this population may be influenced by both external factors and CA’s genetic characteristics.

Mortality from CA infection varies between 30 and 60% [4,17]. This is influenced by multiple factors including the severity of the patient’s underlying disease, characteristics of CA (virulence, drug resistance) among others [18,19]. Our observation of 37.8% mortality is consistent with existing literature, underscoring the importance of recognizing and treating ICAD early. Since colonization is also a major risk factor for development of invasive disease, and nosocomial transmission and outbreaks have been reported, efforts to prevent colonization and nosocomial transmission are important tools in reducing the consequences of CA infection. This is highlighted by our observation of 14% mortality in individuals with CA colonization, underscoring the need to prevent colonization, and when it develops, to identify it early and implement strategies to prevent colonization to progress to invasive disease.

Limitations of the study include the retrospective nature of data collection. Retrospective studies, including our study, are limited by non-standardized data collection and some variables may be missing or incomplete. Intervention decisions are dependent on the individual clinician’s choice and there may be a significant variance from one patient to another. Our study lacked molecular characterization of the CA isolates and environmental cultures to determine the mode of spread. As the study was conducted in Qatar only, it restricts generalizability to the other regions. The heavily skewed demographic data regarding sex and nationality is a result of the fact that Qatari nationals make up only about 15% of the population, with the remainder being expatriate workers residing in Qatar [20]. A large proportion of expatriate workers are single males working in various craft and manual professions. However, with study population and conditions similar in the Middle East region, especially immediately surrounding countries, these results may highlight the interventions needed to combat clonal outbreaks of CA. Strengths of our study include structured chart reviews and inclusion of multiple hospitals within a single healthcare system with the same infection prevention and control protocols.

## Conclusion

In conclusion, while a relatively small proportion of individuals with *C*. *auris* infection develop invasive fungal disease, such development is associated with longer hospital stay and higher mortality. Severely ill persons on mechanical ventilation with any positive *C*. *auris* cultures should be especially monitored for development of invasive fungal disease.
